# Physical Exercise Modulates Brain Physiology Through a Network of Long- and Short-Range Cellular Interactions

**DOI:** 10.3389/fnmol.2021.710303

**Published:** 2021-08-19

**Authors:** Alan Consorti, Irene Di Marco, Gabriele Sansevero

**Affiliations:** ^1^Neuroscience Institute, National Research Council (CNR), Pisa, Italy; ^2^NEUROFARBA, University of Florence, Florence, Italy; ^3^Stella Maris Foundation, Calambrone, Italy

**Keywords:** physical exercise, brain physiology, brain pathology, myocytes, neurons, microglia, neurodegeneration, neurodevelopmental disorders

## Abstract

In the last decades, the effects of sedentary lifestyles have emerged as a critical aspect of modern society. Interestingly, recent evidence demonstrated that physical exercise plays an important role not only in maintaining peripheral health but also in the regulation of central nervous system function. Many studies have shown that physical exercise promotes the release of molecules, involved in neuronal survival, differentiation, plasticity and neurogenesis, from several peripheral organs. Thus, aerobic exercise has emerged as an intriguing tool that, on one hand, could serve as a therapeutic protocol for diseases of the nervous system, and on the other hand, could help to unravel potential molecular targets for pharmacological approaches. In the present review, we will summarize the cellular interactions that mediate the effects of physical exercise on brain health, starting from the factors released in myocytes during muscle contraction to the cellular pathways that regulate higher cognitive functions, in both health and disease.

## Introduction

Among the number of different activities that shape lifestyle, physical exercise is known to elicit various beneficial consequences on health.

At systemic level, it has been shown to exert a positive outcome in different systems: it prevents cardiovascular diseases and osteoporosis, improves glucose metabolism, and decreases the risk of developing cancer (Warburton et al., 2006). Moreover, the remarkable impact of voluntary physical activity on glucose metabolism exerts relevant beneficial effects in the insulin-resistant state (James et al., 1984) and in preventing type 2 diabetes in high-risk individuals (Pan et al., 1997; Kelley and Goodpaster, 1999).

Furthermore, physical exercise induces a wide range of beneficial effects on brain health at different levels: regardless of the specific type of aerobic activity, it reduces anxiety, depression (Sharma et al., 2006), and negative mood (Callaghan, 2004). At the cognitive level, a relationship between aerobic capacity, hippocampal plasticity and memory is widely recognised and accepted (Duzel et al., 2016), and physical exercise improves and maintains cognition in older individuals (Duzel et al., 2016). Studies conducted in animal models have shown that running modulates neurotransmitters, neurotrophin level, neuronal morphology and adult neurogenesis (Voss et al., 2013). Interestingly, running has also been shown to reinstate juvenile-like plasticity in the visual cortex of adult rats through a reduction of the intracortical inhibitory tone (Baroncelli et al., 2012). Very recently, this approach has been successfully applied to restore plasticity also in adult human subjects (Lunghi and Sale, 2015).

It is worth noting that while moderate physical activity is considered a safe practice – World Health Organization suggests 150–300 min of weekly moderate aerobic exercise for adults [World Health Organization (WHO), 2020], the effects of higher intensities on brain physiology are still controversial. Some studies reported that high-dose exercise induces higher increase of neurotrophins than moderate exercise (Marquez et al., 2015); on the other hand, it has also been reported that the exaggerated oxidative stress exerted by exhausting exercise could impair cognitive functionalities in mice (Rosa et al., 2007). In order to avoid possible misunderstandings, all the works here reported deal about moderate aerobic exercise.

Despite the ever-increasing number of studies investigating the effects of physical activity on the brain, the peripheral mechanisms that drive these beneficial events remain unclear. In particular, very little is known about the physiological processes involved in the translation of general muscle activation into the enhancement of brain molecular pathways involved in neural plasticity.

Given the complexity of the cellular and biochemical framework that underlies physical exercise’s output on brain physiology, a reductionistic approach to this issue is not possible and the present work is not aimed to suggest the pivotal role of a particular pathway or cellular network over others; it is rather conceived as a map that untangles some of the numerous ropes that span from muscular activation to brain health. Thus, this review focuses on the cellular interactions that regulate brain physiology in response to physical exercise in health and disease.

## From Periphery to CNS: Different Cellular Interactions Modulate the Effects of Physical Exercise on the Brain

The journey from muscle contractions to neuronal modulation is assembled by numerous routes: the molecules released by peripheral organs are regulated through the somatotropic axis with feedback and feedforward mechanisms, that eventually contribute to environment perception and cognitive functionalities.

### Periphery to CNS

In response to physical exercise, several peripheral organs release a plethora of molecular factors in the bloodstream. Once in the circulatory system, some of these factors can cross the blood-brain barrier (BBB) and enter the brain where they affect the neuronal activity of CNS cells.

Skeletal muscle is one of the major sources of these exercise-induced circulating factors; during prolonged muscle contraction, myocytes produce, and secrete small proteins called myokines that act on various other organs including the heart, liver, pancreas, and the brain (Schnyder and Handschin, 2015). By definition, myokines are cytokines released by skeletal muscles, exerting both an autocrine control on muscle metabolism and a paracrine or endocrine regulation on other body parts (Pedersen et al., 2003). Among them, irisin, a recently discovered myokine (Boström et al., 2012), is getting increasing attention. In skeletal muscle, prolonged physical exercise activates the peroxisome proliferator-activated receptor γ coactivator 1α (PGC-1α) through the 5′ AMP-activated protein kinase (AMPK). In turn, PGC-1α increases the blood concentration of irisin controlling the expression of fibronectin type III domain-containing protein 5 (FNDC5), the transmembrane protein from which irisin is cleaved. In the brain, this myokine exerts an antidepressant-like effect (Wang and Pan, 2016; Siteneski et al., 2018) and confers neuroprotection in animal models (Xia et al., 2017; Noda et al., 2018; Lourenco et al., 2019). Similarly, physical exercise increases the level of cathepsin B that has anti-amyloidogenic and neuroprotective functions (Mueller-Steiner et al., 2006) and whose expression is required for functional spatial memory, proper mood-related behavior, and adult neurogenesis (Moon et al., 2016).

Myocytes are not the only peripheral cell type modulating neuronal activity during locomotion; indeed, hepatocytes produce and secrete molecular factors in response to vigorous exercise as well. The insulin-like growth factor 1 is the earliest identified peripheral factor that mediates the interaction between peripheral and brain cells following physical activity (Carro et al., 2000). IGF-1 belongs to the insulin superfamily, a large group of peptides sharing a highly conserved structural motif consisting of two peptide chains linked by three disulfide bonds (Shabanpoor et al., 2009). Insulin and insulin-like growth factors (IGF-1 and IGF-2) are chiefly known for their role in controlling glucose metabolism peripherally, but they also have various functions in the CNS. In the developing brain, IGF-1 is locally expressed by all cell types; this peptide is indeed necessary for cell proliferation, survival, and differentiation as well as for proper axon guidance and synaptogenesis during CNS formation (Beck et al., 1995; O’Kusky et al., 2000; Cheng et al., 2001; Hodge et al., 2007; Hurtado-Chong et al., 2009; Croci et al., 2011; Fernandez and Torres-Alemán, 2012; for a review). However, the local synthesis of this peptide dramatically drops postnatally and, though IGF-1 is produced by all adult tissues, hepatocytes are the main source of this trophic factor in adulthood (Bartlett et al., 1991; García-Segura et al., 1991; Bondy et al., 1992).

The hepatic expression of IGF-1 is regulated by the growth hormone (GH) synthesized by the somatotropic cells in the pituitary gland (Hartman et al., 1993). Notably, the serum concentration of GH is increased in running trained subjects (Sutton and Lazarus, 1976; Hartman et al., 1993; Kraemer et al., 1993). When released into the serum, IGF-1 is bound by the IGF-binding proteins (IGFBPs), a group of six proteins that modulate the bioavailability of circulating IGF-1 (Allard and Duan, 2018) and transport it to the brain.

Stimulating the release of peripheral factors, physical exercise has a profound influence on both the developing and the adult CNS. To date, the mechanisms by which these messengers modify neuronal activity are incompletely understood; however, accumulating evidence suggests that they may ultimately promote epigenetic changes on the promoters of Brain-derived neurotrophic factor (BDNF) (Gomez-Pinilla et al., 2011), which is a master regulator of neural function (Kowiański et al., 2018). Consistent with this, a recent study (Sleiman et al., 2016) proved that β-hydroxybutyrate, one of the factors secreted by the liver during physical exercise, can increase the expression of this neurotrophin inactivating the histone deacetylases (HDACs), which are a class of enzymes known to decrease BDNF synthesis (Koppel and Timmusk, 2013).

### Brain-Blood Barrier and Brain-Cerebrospinal Fluid Barrier

The neural microenvironment is isolated from plasma by several selective membranes, including the BBB and the blood-cerebrospinal fluid (CSF) barrier (BCSFB). BBB supplies the brain with essential nutrients and mediates the efflux of many waste products (Begley and Brightman, 2003). It plays an important role in the homeostatic regulation of the brain microenvironment necessary for the stable and coordinated activity of neurons (Cserr and Bundgaard, 1984), limits small molecule permeation, regulates large molecular traffic, and also separates peripheral and central neurotransmitters pools (Abbott, 2004). BBB is formed by brain endothelial cells lining the cerebral microvasculature, but neurons, astrocytes, pericytes, extracellular matrix and microvessels are organized into well-structured neurovascular units (Hawkins and Davis, 2005), which are involved in the regulation of cerebral blood flow (Iadecola, 2004). However, the main structures responsible for the barrier properties of the BBB are tight junctions (TJs) (Reese and Karnovsky, 1967), that seal the intercellular cleft between one cell and another.

Blood-brain barrier is not the only barrier dividing the CNS from the periphery, a second interface is formed by the epithelial cells of the choroid plexus (CP) (Wolburg and Paulus, 2010). With the aim of separating blood and CSF compartments, the epithelial cells of the CP and the tanycytes of circumventricular organs (CVO) constitute the BCSFB (Segal, 2000). Similar to BBB, TJs between the CP epithelial cells inhibit paracellular diffusion of hydrophilic substances (Engelhardt and Sorokin, 2009). Compared to BBB, this barrier is leakier (Wolburg and Lippoldt, 2002), but this does not reflect an increase in the bioavailability of substances in the deep brain parenchyma (Spencer et al., 2020). Besides their barrier function, CP epithelial cells are involved in the supply and distribution of peptides into the brain, in the removal of toxic metabolites, in the excretion of xenobiotics (Engelhardt and Sorokin, 2009; Weiss et al., 2009), and in the production of CSF through free access to the blood compartment of the leaky blood vessels (Brown et al., 2004).

TJs act as a “physical barrier” (Abbott et al., 2006, 2010) that precludes the entry into the CNS of a whole series of molecules necessary for its functioning. Only small gaseous molecules, such as O_2_ and CO_2_, and a wide range of lipid-soluble molecules can passively diffuse across the lipid membranes of the epithelial cells (Liu et al., 2004). Most polar molecules cannot diffuse through the BBB thus, all endothelial cells express a large number of solute carrier (SLC) proteins in the membrane (Zhang et al., 2002) that mediate the influx and efflux of these molecules. Carrier-mediated transport (CMT) allows the entry of essential nutrients (i.e., glucose) into the brain and the elimination of metabolic waste.

Large molecules such as peptides and proteins also require a transport mechanism to cross the BBB: they can pass through the endothelial cell membranes via the specific receptor-mediated transcytosis (RMT), or by the less specific adsorptive-mediated transcytosis (AMT) (Pardridge, 2003; Wolka et al., 2003; Abbott et al., 2006). In particular, IGF-1 and IGF-2, undergo RMT across the BBB via separate type 1 and type 2 IGF receptors (IGF1R and IGF2R) (Pardridge, 2007). In the circulatory system, the great majority of serum IGF-1 forms a 150 kDa complex along with the acid-labile subunit (ALS) and the IGFBP3 (Delafontaine, 1995), i.e., the IGFBP with the highest affinity to IGF-1. Blood released IGF-1 can then enter the CNS via two distinct gateways: it can enter the CSF through the CP or the brain parenchyma through the BBB (Carro et al., 2005; Nishijima et al., 2010). These two structures are indeed well suited to transport serum IGF-1: the CP epithelium, the end-feet glial cells and the brain vessels in the BBB express high amounts of IGF-1Rs (García-Segura et al., 1991; Marks et al., 1991). The CSF concentration of IGF-1 linearly depends on the systemic level of this trophic factor; an observation suggesting that IGF-1 is constitutively transported into the CSF through the CP (Carro et al., 2000, 2005). This direct passage of IGF-1 into the CSF occurs through a mechanism involving the multiligand receptor megalin expressed in the CP epithelium (Carro et al., 2005). In parallel, serum IGF-1 is carried in the brain parenchyma via an activity-dependent mechanism; the entrance of IGF-1 through the BBB is guided by a process initiated by glutamate release at active brain regions (Nishijima et al., 2010). The release of glutamate by active synapses leads to Ca^2+^ influx in astrocytes with the subsequent release of prostaglandin E2, arachidonic acid derivatives, NO and ATP. These diffusible mediators induce local vasodilation increasing the BBB permeability to serum IGF-1, oxygen, and glucose. Furthermore, several of these same mediators activate the matrix metalloproteinase 9 (MMP9) that cleaving IGFBP3 releases bioavailable IGF-1. The MMP9 activity, therefore, raises the amount of serum IGF-1 able to bind the IGF-1Rs, which are locally expressed by the BBB (Nishijima et al., 2010). Crossed the BBB, IGF-1 can directly interact with active neurons or can be indirectly transported to active neurons by the end-feet glial cells. Upon entry in the adult brain, IGF-1 controls cellular homeostasis, modulates synaptic plasticity, promotes adult neurogenesis, and counteracts neurodegeneration (Fernandez and Torres-Alemán, 2012).

A great number of CNS pathologies (including brain injury, stroke, multiple sclerosis, epilepsy, Parkinson’s disease, and Alzheimer’s disease) cause TJ dysfunctions (Förster, 2008) that, increasing BBB permeability, leading to the entry of neurotoxic substances into the brain. Lifestyle and bad eating habits also influence the functioning and integrity of the BBB. Metabolic syndrome, insulin resistance, type 2 diabetes, arterial hypertension, dyslipidaemia, and obesity cause low-grade inflammation due to increased blood levels of pro-inflammatory cytokines including Interleukin-1β,-6 (IL-1β,-6) and tumour necrosis factor-α (TNF-α) and the CNS response to inflammation could lead to endothelial cell damage which increases BBB permeability (Kim et al., 2012). It has been shown that moderate physical exercise can maintain endothelial health (D’Alessio, 2004; Middlebrooke et al., 2005). Furthermore, exercise induces a reduction in C-reactive protein (CRP), IL-1, IL-6, interferon-γ (INF-γ) levels in coronary artery disease patients (Goldhammer et al., 2005), demonstrating its anti-inflammatory effects. A reduction of TNF-α and IL-6 blood levels has been observed also in a group of healthy elderly women after 14 weeks of exercise (Chupel et al., 2018). Diabetes mellitus and obesity are frequently associated with oxidative stress (Houstis et al., 2006) that refers to elevated intracellular levels of reactive oxygen species (Schieber and Chandel, 2014) which produce a loss in TJs integrity (Baeten and Akassoglou, 2011) causing a direct damage on BBB. In animal model of obese type 2 diabetes regular and moderate physical exercise reduce oxidative stress (Teixeira-Lemos et al., 2011). Therefore, counteracting obesity, physical exercise indirectly protects the BBB. In short, physical exercise seems to be an important therapeutic resource that protects and repairs the BBB which in turn maintains homeostasis in the cerebral microenvironment.

### Hippocampus

The impact exerted on the CNS by exercise has been extensively studied in the hippocampus, a brain region embedded in the medial temporal lobe with a major role in learning and memory (Bird and Burgess, 2008).

In human subjects, moderate-intensity physical activity was correlated to increased hippocampal size, hippocampal blood flow, and memory (Erickson et al., 2011; Steventon et al., 2020). Likewise, exercise has been shown to improve spatial navigation (Ang et al., 2006; Aguiar et al., 2011), object recognition (Bechara and Kelly, 2013) and contextual fear memory (Greenwood et al., 2009) in rodents. These memory improvements appear to be mediated by the genesis of new CNS cells in the subgranular zone (SGZ), a stem-cell niche that lies between the hilus and the granule cell layer in the hippocampal dentate gyrus (Gonçalves et al., 2016). Physical exercise indeed promotes adult neurogenesis and newborn cell survival (Van Praag et al., 1999a,b) through a mechanism that could be mediated by BDNF expression (Liu and Nusslock, 2018). In fact, growing evidence supports the notion that BDNF is required for proper neuronal differentiation and survival in adulthood (Lee et al., 2002; Scharfman et al., 2005; Chan et al., 2008; Waterhouse et al., 2012). Moreover, this enhancement of neurogenesis is an exclusive process to the SGZ; physical exercise influences the production of new cells in the hippocampal formation but not in the subventricular zone, which is one of the two major stem-cell niches in the adult brain along with the SGZ (Brown et al., 2003).

Hippocampal neurogenesis is intimately connected with angiogenesis. Newborn cells cluster in striking proximity to blood vessels (Palmer et al., 2000) that are integral constituents of stem-cell niches since the vasculature serves not only as a conduit for nutrients, but also conveys signaling molecules that modulate stem-cell differentiation (Licht and Keshet, 2015). Physical exercise shapes the neurovascular interface augmenting the vascular density in the granular layer of the dentate gyrus. Vascular endothelial growth factor (VEGF) originating from outside the BBB represents an essential player in this angiogenic response to exercise. Following acute muscle contractions, skeletal myofibers release VEGF in circulation (Fabel et al., 2003; Rich et al., 2017). Once in the cortex, VEGF stimulates both the proliferation of neural cell precursors and the perfusion of blood capillaries in the SGZ (Jin et al., 2002; Fabel et al., 2003; van Praag et al., 2005; Ding et al., 2006; Clark et al., 2009; Rich et al., 2017). In agreement with this hypothesis, the peripheral injection of a VEGF antagonist abolishes exercise-induced SGZ neurogenesis but is ineffective in suppressing baseline neurogenesis (Fabel et al., 2003). However, according to recent lines of research, the local VEGF production by CNS resident cells might itself instruct the SGZ neurovascular interface. During physical exercise, hypoxic conditions favour the pyruvate conversion into L-lactate in skeletal muscles. This metabolite is then secreted in the bloodstream and transported in the CNS through the monocarboxylate carriers (Proia et al., 2016). In the dentate gyrus, L-lactate increases local VEGF release activating the lactate receptor – hydroxycarboxylic acid receptor 1 – (HCAR1) expressed by the perivascular pial and pericyte-like cells (Morland et al., 2017).

By promoting the genesis of granular cells, physical exercise modulates synaptic plasticity in the hippocampus. Adult newborn cells acquire the properties of completely mature granule cells and are functionally integrated into the hippocampal network by 4 weeks (Van Praag et al., 2002). During this process, newborn cells exhibit a narrow time window of enhanced plasticity that depends on the transient expression of the NR2B subunits of N-methyl-D-aspartate receptors (NMDAR) early (4–6 weeks) post-mitosis (Snyder et al., 2001; Ge et al., 2007). Long-term potentiation (LTP) is consistently enhanced in hippocampal slices of running mice as compared to sedentary animals. This increase in synaptic plasticity is tightly connected with running-induce SGZ neurogenesis; changes in LTP properties can be detected only in the dentate gyrus, where new granule cells born, and not in other hippocampal regions (Van Praag et al., 1999b; Farmer et al., 2004). Furthermore, adult-born cells modify not only the functional properties of the dentate gyrus but also rewire the hippocampal circuitry. Indeed, physical exercise reshapes the local innervation to newborn neurons and increases the distal projections coming to these cells from subcortical and cortical regions (Vivar et al., 2016; Sah et al., 2017).

### Cortical Areas

Cortical areas are the headquarters of higher cognitive functions that allow us to respond adaptively to a constantly changing environment, for this reason these areas are highly influenced by physical activity, which represents a way to interact with the environment. Exercise increases cortical thickness in older individuals (Colcombe et al., 2006) and modulates neuronal protein expression via epigenetic regulation (Abel and Rissman, 2013; Kashimoto et al., 2016). Interestingly, exercise protects microglial cells from age-dependent loss of functionality, a fact that, in turn, improves cognitive functions in elderly subjects (Mela et al., 2020).

It is worth noticing that, locomotion is also able to directly modulate specific cortical areas: from an evolutionary point of view, the integration of motor coordination and sensory information -visual, auditory, and tactile (Lee et al., 2013; Dipoppa et al., 2018; Ayaz et al., 2019; Yavorska and Wehr, 2021) – is a process that is necessary for fundamental skills required for surviving such as foraging and threats escape.

The visual cortex represents, since the 1960s, the prime model to study the complex interaction between external cues – such as physical exercise – and cellular reorganization of neural circuits. The proper maturation of the visual system is a phenomenon strictly dictated by the mutual interaction between genetic programs and plasticity processes driven by environmental experience. Postnatal exposure to an enriched environment, a condition of enhanced physical exercise and sensory stimulation, conspicuously accelerates visual system development (Sale et al., 2004). A number of data now support the hypothesis that this acceleration is partly elicited by the increased amount of serum IGF-1 in enriched animals, which experience high levels of motor stimulation. Such hypothesis is supported by experiments showing that the exogenous supply of JB1 (an IGF-1R antagonist) or anti-IGF-I antiserum completely prevents this acceleration (Ciucci et al., 2007; Landi et al., 2009). The faster maturation of the visual system might be caused by the IGF-1 interaction with different pathways. Indeed, the increased presence of IGF-1 enhances BDNF expression, stimulates retinal development, promotes a precocious maturation of the GABAergic interneurons and a precocious decrease in the NKCC/KCC2 ratio (Sale et al., 2007; Baroncelli et al., 2017). Interfering with all these pathways, IGF-1 can eventually prompt the premature visual system development observed at behavioral, electrophysiological, and molecular level.

Number of studies are needed to evaluate whether other exercise-related molecules extend their action to the visual system. Nevertheless, a number of researches provided evidence that physical exercise acts on visual neurons promoting the release of different neuromodulators in the primary visual cortex; in particular, locomotion increases the amount of serotonin and acetylcholine (Jacobs and Fornal, 1999). In the CNS, serotonin is almost exclusively released by the serotoninergic neurons located in the raphe nuclei, which are a cluster of nuclei in the brain stem (Hornung, 2003). Neurotransmission of serotonin is involved in structural and functional remodeling of visual cortical circuits and is considered one of the non-visual neurochemical bases of attention, arousal, and motivation (Gu, 2007). Serotoninergic neurons produce serotonin through a process that is limited by the plasma level of tryptophan, a large neutral amino acid from which serotonin is synthesized (Russo et al., 2007; Höglund et al., 2019). Tryptophan is transported through the BBB via a transporter carrier shared by all the large neutral amino acids; therefore, tryptophan’s entry in the CNS does not depend on its plasma concentration *per se*, but on its plasma concentration with respect to other large neutral amino acids since they all compete for the same transporter (Fernstrom, 2005; Markus, 2008; Höglund et al., 2019). During physical exercise, the branched-chain amino acids – a subset of the large neutral amino acids – are transported into the muscles (Patrick and Ames, 2015). Consequently, physical exercise increases tryptophan transport into the brain decreasing the competition for the BBB transporter.

On the other hand, the effect of acetylcholine in shaping the circuitry of the visual cortex has been extensively studied in the last few years (Sugihara et al., 2016). The primary visual cortex receives direct input from the nucleus of the diagonal band of Broca, a cholinergic centre in the basal forebrain (Fu et al., 2014), specifically from the mesencephalic locomotor region (MLR), a midbrain region associated with the ascending reticular activating system described by Moruzzi and Magoun (1949). During locomotion, the enhanced activity of the MLR increases visual cortical responsiveness via a mechanism involving the vasoactive intestinal peptide (VIP) and the somatostatin (SST) positive cells, which are two of the major classes of inhibitory interneurons. To dissect this cellular mechanism, Fu et al. (2014) imaged calcium responses of these interneurons in awake mice free to run on a treadmill. They found that neural activity of VIP neurons is significantly increased during locomotion even in the absence of visual stimulation; SST neurons were instead inhibited by locomotion. These observations suggest the involvement of a circuit in which VIP neurons trigger the inhibition of SST neurons. Therefore, the activation of VIP cells boosts cortical excitation releasing pyramidal cells from the inhibition exerted by SST neurons, a mechanism termed disinhibition. VIP interneurons are activated by the basal forebrain through nicotinic acetylcholine receptors (nAChRs) (Alitto and Dan, 2012). Consistently, local infusion of nAChRs antagonists greatly reduces the response of VIP cells to locomotion, pinpointing acetylcholine as a crucial mediator of physical exercise in the primary visual cortex (Fu et al., 2014).

## Physical Exercise as a Potential Therapeutic Tool for CNS Pathologies

**FIGURE 1:**
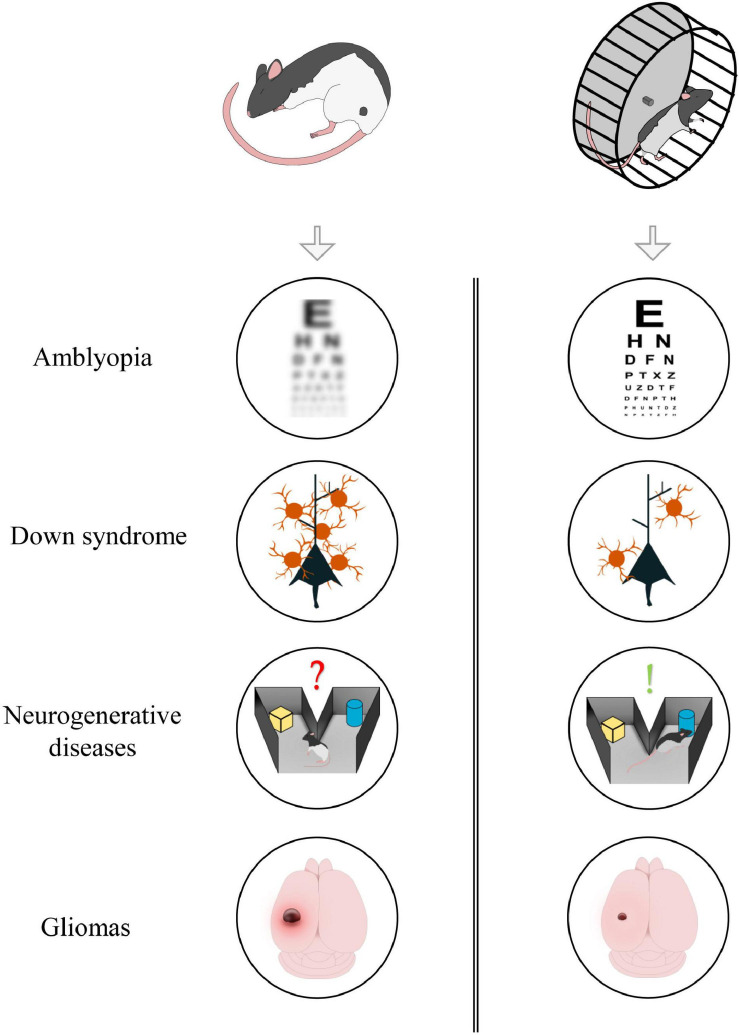
Aerobic exercise could exert beneficial effects in different forms of neuropathology. From top to bottom: exercise induces visual recovery in adult amblyopic subjects; it reduces GABAergic innervation in animal model of Down Syndrome; it improves memory formation and consolidation in neurodegenerative diseases; it contributes in reducing tumoral mass in glioma-bearing mice.

Physical exercise has a marked influence on several physiological processes; indeed, general myocyte contraction can, indirectly, control adult neurogenesis, cortical excitability, and the release of different neuromodulators such as BDNF. Given its multifactorial action, exercise, and its downstream cellular pathways, could represent potential targets for preventing or reversing the symptoms of those pathologies in which neuroprotection, plasticity and transmission are defective (Figure 1). In the following section, we describe amblyopia, Down syndrome, neurodegenerative diseases, and glioma as explicative cases.

### Amblyopia

Amblyopia, with an incidence of 1–5%, is the prevalent monocular impairment in the world human population (Holmes and Clarke, 2006). The central physiological mechanism in amblyopia is considered inter-ocular suppression, which is the empowering of the inputs coming from the apparently healthy eye at the expense of those coming from the amblyopic eye (Antonini et al., 1999). This suppression is evident at the level of the primary visual cortex and appears to be mediated by the inhibitory circuitry (Sengpiel et al., 2005).

Amblyopia, if not promptly treated during childhood, leads to severe visual impairments including loss of visual acuity and defective stereopsis. Occlusion therapy, i.e., the temporary exclusion of the healthy eye from visual activity by means of an eye patch, completely reverses amblyopia when performed during the critical period for binocular vision (Loudon and Simonsz, 2005). Nevertheless, amblyopia is an almost untreatable disease in adulthood; the occlusion therapy is indeed completely ineffective in adult subjects due to the dramatic decline in cortical plasticity caused by the maturation of different plasticity-limiting factors as the GABAergic and the cholinergic systems (Fagiolini and Hensch, 2000; Morishita et al., 2010).

Amblyopia can be artificially induced in animal models depriving one eye of visual stimuli through a long-term lid suture. Several studies have proved physical exercise as an effective strategy to treat amblyopia in adult rodents. Employing adult amblyopic rats, it has been recently shown that voluntary physical exercise under binocular conditions promotes a full and long-lasting recovery of both visual acuity and depth perception (Sansevero et al., 2020). Moreover, physical exercise can also promote amblyopia recovery in humans; indeed, it has been shown that a short-term occlusion of the amblyopic eye coupled with moderate physical activity improve visual acuity and stereo-sensitivity in adult patients (Lunghi et al., 2019). Notably, the exercise-induced factors may be directly involved in promoting the beneficial effects exerted by physical exercise; pharmacological interventions enhancing IGF-1, serotonin or serotonin transporters can counteract visual impairments in adult amblyopic rodents even in the absence of intense exercise (Vetencourt et al., 2008; Maya Vetencourt et al., 2011; Maya-Vetencourt et al., 2012). However, a thorough analysis is still needed to understand whether these molecular factors promote amblyopia recovery increasing BDNF expression. BDNF itself, indeed, can be successful in eliciting visual function recovery in adult animals (Sansevero et al., 2019).

It is worth noting that short-distance cellular interactions also play a crucial role in promoting amblyopia recovery in physical active animals. Recent studies have indeed demonstrated that activation of the VIP-SST disinhibitory circuit promoted, during locomotion, by cholinergic afferents originating from the mesencephalic locomotor region, induces a complete recovery of responsiveness in both excitatory and inhibitory neurons in the primary visual cortex (Kaneko and Stryker, 2014; Sansevero et al., 2020). In this scenario, physical exercise promotes amblyopia recovery releasing the excitatory neurons from excessive levels of inhibition exerted by SST interneurons.

Based on the above-reported studies, a complex and interactive model could be postulated in which both long- and short-range interactions contribute to visual function recovery in running amblyopic animals; with long-distance cellular interaction controlling the expression of pro-plastic genes – like bdnf – and short-distance cellular interaction increasing cortical responsiveness through the release of pyramidal neurons from the local inhibition.

### Down Syndrome

Down syndrome (DS), a developmental disorder elicited by the partial or total triplication of the chromosome 21 (Hsa21), is the most common genetic cause of intellectual disability in humans, with an incidence ranging from 1 in 700 to 1 in 1000 live births (Roizen and Patterson, 2003). People with DS exhibit major cognitive deficits in learning and memory along with a number of moderate to severe impairments in motor function, language, and sensory processing (Contestabile et al., 2010; Dierssen, 2012).

The last two decades have brought the development of several mouse models with DS-related features (Gupta et al., 2016; Herault et al., 2017). To date, Ts65Dn is one of the most commonly used and best-studied model of DS. Ts65Dn is characterized by the triplication of the distal segment of Mm16, the mouse chromosome harbouring the largest syntenic region of homology to Hsa21 (Davisson et al., 1990; Duchon et al., 2011). Ts65Dn mice recapitulate the main hallmarks of DS phenotypes. Ts65Dn indeed displays impairments in learning and memory (Hunter et al., 2003), motor dysfunction (Costa et al., 1999), visual deficits (Scott-McKean et al., 2010) and several anatomical alterations including craniofacial dysmorphology (Richtsmeier et al., 2002), hippocampal hypocellularity (Guidi et al., 2008), and impaired neurogenesis (Lorenzi and Reeves, 2006). The Ts65Dn phenotype correlates with major functional deficits in synaptic plasticity, particularly in the hippocampus where the possibility to induce LTP is drastically compromised (Siarey et al., 1997, 1999). This LTP failure is largely determined by alterations in the GABAergic system (Kleschevnikov et al., 2004; Costa and Grybko, 2005). Indeed, marked morphological and functional changes have been detected in the GABAergic circuitries of both the cerebral cortex and the hippocampus of Ts65Dn mice (Belichenko et al., 2009; Chakrabarti et al., 2010; Best et al., 2012; Martin et al., 2020). Therefore, this large body of evidence has now led to the notion that the excessive level of cortical inhibition is the major functional impairment in this DS model (Best et al., 2007). Accordingly, pharmacological interventions targeting the GABAergic system can completely reverse the defects displayed by Ts65Dn mice (Fernandez et al., 2007), confirming the prime role of overinhibition in DS pathogenesis (Baroncelli et al., 2011; Contestabile et al., 2017).

Physical exercise emerged as an appealing strategy to ameliorate Ts65Dn mice conditions, notably for its role in restoring the proper excitatory/inhibitory balance in the CNS (Sale et al., 2014); an effect largely ascribed to the activation of an interaction network between specific GABAergic cell subtypes (Stryker, 2014). Complex sensory-motor stimulation, including a high level of voluntary physical exercise, can indeed restore spatial navigation, hippocampal plasticity, and brain development in Ts65Dn promoting a reduction in the GABA transmission paralleled by an increase in BDNF expression (Baroncelli et al., 2011; Begenisic et al., 2015). Strikingly, the overexpression of this neurotrophic factor has been extensively associated with reduced inhibition in the adult brain (Sale et al., 2010; Baroncelli et al., 2011). Recent studies have also shown that physical exercise *per se* might exert a positive effect on the DS phenotype promoting recovery of learning and cognitive performances in Ts65Dn mice (Llorens-Martín et al., 2010; Parrini et al., 2017). In human subjects with DS, exercise has been shown to improve physical fitness (Rimmer et al., 2004; Dodd and Shields, 2005; Mendonca et al., 2011). Nonetheless, little is known about the effects of physical exercise on cognitive functions. Recent studies, however, seem to suggest that exercise might also improve executive functions in DS individuals (Pape et al., 2021). Likewise, the exercise-related factors might represent promising targets for therapeutic application to DS since they could be particularly effective in reducing overinhibition by increasing the neuronal expression of BDNF. Interestingly, a recent work showed that either physical exercise or a BDNF-mimetic pharmacological intervention can rescue cognitive functions and synaptic plasticity in Ts65Dn mice (Parrini et al., 2017). Moreover, it is noteworthy to point out that pharmacological strategies based on exercise-related factors can circumvent the major throwback of BDNF administration, i.e., the impossibility for this neurotrophic factor to efficiently cross the blood-brain barrier when delivered peripherally (Nagahara and Tuszynski, 2011).

### Neurodegenerative Diseases

Neurodegenerative diseases (NDs) are a heterogeneous group of pathologies resulting from the progressive loss of selective neuronal types in specific CNS regions. Major NDs include Alzheimer’s disease (AD), Parkinson’s disease (PD), and dementia with Lewy bodies (DLB) (Erkkinen et al., 2018). Ageing is nowadays considered the primary risk factor for neurodegeneration; most NDs occur after the fourth decade of life and their prevalence increases with increasing age (Wyss-Coray, 2016; Hou et al., 2019).

Although the clinical symptoms are diverse depending on the core population of neurons involved in the neurodegenerative process, NDs can be broadly classified as: (1) diseases causing cognitive decline, dementia, and alterations in higher cortical functions; (2) diseases characterized by motor dysfunctions including hyperkinetic and hypokinetic movements (Kovacs, 2018). However, in spite of their heterogeneity in clinical symptoms, numerous NDs share common pathological features: deposition of misfolded proteins, oxidative stress, apoptosis, and neuroinflammation (Dugger and Dickson, 2017; Marsh, 2019).

In search of candidate molecular and cellular mechanisms underlying neurodegeneration, different mouse strains have been generated to model NDs exploiting either genetically based or pharmacologic-based strategies (Dawson et al., 2018; Fisher and Bannerman, 2019). Accumulating evidence shows that physical exercise and exercise-related factors can confer neuroprotection in mouse models of NDs. In recent years, physical exercise emerged as a promising non-invasive strategy to ameliorate neuronal loss, memory impairments, oxidative stress, neuroinflammation, and motor dysfunctions (Souza et al., 2013; Hüttenrauch et al., 2016; Do et al., 2018; Klemann et al., 2018; Palasz et al., 2019). Remarkably, exercise seems to reverse the genetic pattern of inflammation and apoptotic markers set in motion by the progression of NDs (Hüttenrauch et al., 2016; Do et al., 2018) probably through epigenetic mechanisms (Ferioli et al., 2019). A large number of studies has also analyzed the neuroprotective action of specific exercise-released factors. Lourenco et al. (2019) recently reported that irisin levels are reduced in AD mice. Moreover, these authors showed that the peripheral overexpression of irisin attenuates synaptic and memory impairments and that the blockade of either peripheral or brain irisin conversely hampers the neuroprotective effects of physical exercise in AD model. The effect of another myokine, cathepsin B, on AD is instead controversial. According to some studies, cathepsin B can exert neuroprotective actions and promote learning and memory in AD mice (Mueller-Steiner et al., 2006; Embury et al., 2017); on the contrary, other studies have associated this myokine with AD onset and amyloid plaque accumulation (Cataldo and Nixon, 1990; Hook et al., 2005). In addition to myokines, IGF-1 and β-hydroxybutyrate can mitigate the phenotype of various mouse models of NDs (Carro et al., 2006; Lim et al., 2011). IGF-1 can serve as a protective agent against neuroinflammation modulating the activation of CNS resident cells. This growth factor indeed attenuates the inflammatory response of astrocytes (Bellini et al., 2011), decreases the expression of pro-inflammatory cytokine (Park et al., 2011), and it might also repress neurotoxic microglia (Grinberg et al., 2013; Rodriguez-Perez et al., 2016).

The results obtained in mouse models of NDs encourage stronger efforts in the application of physical exercise to human patients; in particular, exercise-related factors may be promising pharmacological targets to counteract, or at least to prevent, the progression of NDs.

### Gliomas

Gliomas (GLs) are primary brain tumours that arise from glial or precursor cells and represent approximately 25.1% of all CNS tumours and 80.8% of malignant tumours. Although the 5-year survival rate for non-malignant primary brain tumours is 91.7%, for malignant tumours this rate drops to 36% and the worst prognosis is for patients diagnosed with glioblastoma (the most malignant GLs) whose 5-year survival rate is 7.2% (Ostrom et al., 2020).

Researchers are paying more and more attention to the glioma microenvironment and to the crosstalk between glioma and peritumoral cells. Glioma cells secrete an aberrant quantity of the excitatory neurotransmitter glutamate, which leads peritumoral neurons to an excitotoxic death via hyperactivation of NMDAR and α-amino-3-hydroxy-5-methyl-4-isoxazole propionic acid receptors (AMPAR) in peritumoral neurons that in turn can make it easier for glioma to invade nearby CNS parenchyma (Ye and Sontheimer, 1999). Concurrently, the autocrine activation of NMDAR and AMPAR promotes glioma invasion (Lyons et al., 2007; Piao et al., 2009; Nandakumar et al., 2019). Another line of research explores the ability of glioma cells to create contact with neurons forming the neuron-glioma synapses (NGS). Under normal conditions, there are synaptic contacts between neurons and normal oligodendroglial precursor cells (OPCs) (Bergles and Jahr, 2000). Recently, Venkatesh et al. showed that the characteristics of NGS are similar to synapses formed between neurons and normal OPCs (Venkatesh et al., 2019). NGS, whose activity is mediated by AMPA receptors, promotes glioma progression (Venkatesh et al., 2019); indeed, AMPA antagonism reduces the glioma cell proliferation (Venkataramani et al., 2019). Furthermore, the increase in glutamate release stimulates peritumoral neurons to release BDNF and Neuroligin 3 (NLGN3) that in turn stimulate the glioma cells to proliferation and infiltration and sustained NGS formation (Venkatesh et al., 2015, 2017, 2019; Venkataramani et al., 2019).

Physical exercise might represent a safe, easy, and side-effect-free treatment for GLs. Tantillo et al. (2020) have recently examined the effect of physical exercise in mice injected with tumour cells (GL261) into the primary motor cortex. Compared to the sedentary group, the trained group showed a reduction in glioma cell proliferation and a slowdown in the emergence of motor deficits, while no difference in tumour volume emerged between the two groups (Tantillo et al., 2020). Consistent with this evidence, other studies have shown that physical exercise suppresses tumour growth and can decrease the risk of a number of cancers such as those of the colon, breast and endometrium (Booth et al., 2012). Hence, physical exercise could be used as a protective factor as well as an adjuvant treatment. Indeed, it has been shown that physical exercise, performed during the temozolomide treatment, prolongs the survival of glioma-mice (Lemke et al., 2016). Consistently, physical exercise can also ameliorate the quality of life in human patients (Cordier et al., 2019).

Exercise increases IGF-1 levels both in the periphery (Carro et al., 2001) and the brain (Carro et al., 2000) and increases the BDNF expression (Vaynman and Gomez-Pinilla, 2005) in specific brain areas. These exercise-related factors may be responsible for reducing or slowing down the progression of glioma modifying the glioma microenvironment (Garofalo et al., 2015, 2017). BDNF stimulates the production of Interleukin-15 (IL-15) in the brain of glioma-mice, and IL-15, in turn, stimulates natural kill (NK) cells to produce Interferon-gamma (IFN-γ) that affects the phenotype of myeloid cells, promoting the transition to an anti-tumour state (Garofalo et al., 2017). Aerobic respiration, which occurs in the mitochondria of eukaryotic cells, generates energy and, as a result of this oxidative metabolism, several reactive species are produced [among which reactive oxygen species (ROS) is the most abundant]. But, when the physiological balance between production and elimination of reactive species is broken, oxidative stress is generated. Oxidative stress is related to a wide variety of human diseases, including cancer (Sosa et al., 2013). Physical exercise promotes brain function, increases the resistance against oxidative stress and facilitates recovery from oxidative stress (Radak et al., 2013; Accattato et al., 2017).

Further studies are needed to better understand the molecular mechanisms and the cellular interactions underlying the efficacy of physical exercise as a therapy for GLs. Nevertheless, the obtained results suggest that physical exercise might be a valid and effective strategy, not only in slowing down the progression of glioma but also in improving the life quality of GLs patients.

## Conclusion

**FIGURE 2:**
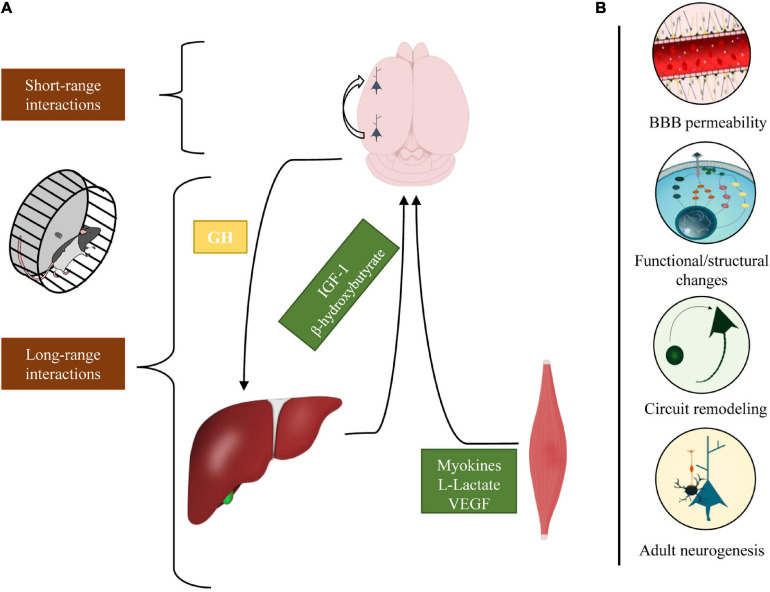
Schematic representation of the different cellular interactions engaged by physical exercise. **(A)** In response to physical exercise, multiple peripheral organs release molecular factors that modulate different neurophysiological processes. The brain, in turn, regulates the inflow of these factors through the somatotropic axis (long-range interactions). Concomitantly, exercise activates intra-cortical neuronal circuits that enhance perceptual and cognitive functions (short-range interactions). **(B)** Physical exercise exerts various effects on the brain modulating BBB permeability, eliciting functional/structural changes, stimulating neurogenesis, and shaping cortical circuits.

We analyzed the impact that physical exercise exerts on brain activity (Figure 2). The work reviewed in this article has shown that physical exercise and brain health are tightly related; indeed, the interplay between brain and muscle activation begins with long-range communications: motoneurons activate myocytes that, in turn, release a large number of factors in the circulation that may reach the brain and regulate the somatotropic axis. On the other hand, in different brain regions, exercise promotes the activation of specific short-range cellular interaction that mediates disparate processes, encompassing from sensory integration to plastic and metabolic changes.

The first consideration, that should emerge from the present work, is that a complete comprehension of the mechanisms that act during exercise on the brain and vice-versa is far from being reached. We marked some boundaries of this tangled network, and we hope they could represent a helpful starting point for future research. As a consequence of that, from a research point of view, the application of multiple experimental approaches but also the commitment of scientists with different formations and perspectives is necessary to develop a field that engages many aspects of general physiology.

Finally, it is important to mention the enormous impact that lifestyle exerts on physiological and pathological brain processes. Effective treatments for most neurodegenerative and neurodevelopmental disorders are still lacking, a fact that entails a huge economic impact on healthcare systems and tremendous consequences on the life quality of families that face such conditions. Given this situation, maintaining an active life, both intellectually and physically, remains one of the few compelling strategies to prevent cognitive decline in the elderly.

## Author Contributions

GS and AC conceived the work and wrote the manuscript. ID wrote the manuscript. All authors contributed to the article and approved the submitted version.

## Conflict of Interest

The authors declare that the research was conducted in the absence of any commercial or financial relationships that could be construed as a potential conflict of interest.

## Publisher’s Note

All claims expressed in this article are solely those of the authors and do not necessarily represent those of their affiliated organizations, or those of the publisher, the editors and the reviewers. Any product that may be evaluated in this article, or claim that may be made by its manufacturer, is not guaranteed or endorsed by the publisher.
